# Retinal eccentricity modulates saliency-driven but not relevance-driven visual selection

**DOI:** 10.3758/s13414-024-02848-z

**Published:** 2024-01-25

**Authors:** Mieke Donk, Elle van Heusden, Christian N. L. Olivers

**Affiliations:** 1 Department of Experimental and Applied Psychology, Vrije Universiteit Amsterdam, Van der Boechorststraat 5-7, 1081 BT Amsterdam, the Netherlands; 2 Institute Brain and Behavior (iBBA), Amsterdam, the Netherlands

**Keywords:** Eye movements, Visual search, Saliency, Relevance, Retinal eccentricity

## Abstract

Where we move our eyes during visual search is controlled by the relative saliency and relevance of stimuli in the visual field. However, the visual field is not homogeneous, as both sensory representations and attention change with eccentricity. Here we present an experiment investigating how eccentricity differences between competing stimuli affect saliency- and relevance-driven selection. Participants made a single eye movement to a predefined orientation singleton target that was simultaneously presented with an orientation singleton distractor in a background of multiple homogenously oriented other items. The target was either more or less salient than the distractor. Moreover, each of the two singletons could be presented at one of three different retinal eccentricities, such that both were presented at the same eccentricity, one eccentricity value apart, or two eccentricity values apart. The results showed that selection was initially determined by saliency, followed after about 300 ms by relevance. In addition, observers preferred to select the closer over the more distant singleton, and this central selection bias increased with increasing eccentricity difference. Importantly, it largely emerged within the same time window as the saliency effect, thereby resulting in a net reduction of the influence of saliency on the selection outcome. In contrast, the relevance effect remained unaffected by eccentricity. Together, these findings demonstrate that eccentricity is a major determinant of selection behavior, even to the extent that it modifies the relative contribution of saliency in determining where people move their eyes.

## Introduction

We can only direct our eyes at one thing at a time. What then determines what we look at? Research on visual selection has focused on several major factors (Awh et al., 2012; Luck et al., 2021; Wolfe & Horowitz, 2017), but has largely ignored the fact that visual processing changes from the fovea to the periphery. Here we determine how differences in the eccentricity of competing stimuli affect visual selection.

Theories of visual selection commonly identify two major sources of control: saliency-driven and relevance-driven control. In saliency-driven control, the eyes are guided to prioritize those locations that are distinct in terms of local feature contrast (Itti & Koch, 2000; Theeuwes, 1992, 1994). In relevance-driven control, selection is biased to prioritize locations containing stimulus features that correspond to the observer’s control settings as defined by intentions, instructions, or task (Folk et al., 1992). Even though the gaze can also be subject to other influences (for further sources, see: Awh et al., 2012; Jiang & Sisk, 2019; Luck et al., 2021; Wolfe & Horowitz, 2017), saliency and relevance are typically considered to be key factors, and the question of which is more powerful in determining selection has been a matter of much debate over the past 30 years (Luck et al., 2021). Importantly, research has shown that the extent to which selection behavior is driven by saliency or relevance is critically dependent on the time a response is triggered (Dombrowe et al., 2010; Donk & van Zoest, 2008, 2011; Hunt et al., 2007; Schutt et al., 2019; van Heusden et al., 2022; van Zoest et al., 2004; van Zoest & Donk, 2005, 2008). While saliency-driven effects occur early in time but are transient (e.g., Donk & van Zoest, 2008), relevance-driven effects arise later and persist longer throughout the course of a trial (e.g., van Zoest & Donk, 2008).

Very few studies have investigated how eccentricity affects selection. A number of studies (e.g., Carrasco et al., 1995; Carrasco & Yeshurun, 1998; Staugaard et al., 2016; Wolfe et al., 1998) have shown that in visual search reaction time increases with a target’s retinal eccentricity, suggesting that more eccentric items require more time to be selected. However, none of these studies separated saliency-driven and relevance-driven selection. In a recent study (van Heusden et al., 2021), we did investigate how the eccentricity of two competing stimuli affects these two types of selection behavior. In this study, participants were asked to make an eye movement to a prespecified target singleton in the presence of an irrelevant distractor singleton. The target and distractor were simultaneously presented at one of three possible retinal eccentricities and either the target or the distractor was the most salient singleton in the display. We then analyzed the landing position of the first eye movements (on target or on distractor) as a function of the saccade latency, allowing us to assess how the effects of saliency and relevance vary across time. The results replicated earlier findings showing that short-latency eye movements were primarily driven by saliency, whereas long-latency eye movements were driven by relevance. Moreover, as eccentricity increased, the latency of both saliency-driven and relevance-driven eye movements increased, indicating that the eccentricity of the singletons shifted the time courses of both types of control. Importantly, the overall proportions of saliency-driven and relevance-driven eye movements (averaged across all saccade latencies) remained constant across eccentricity. This suggests that, while eccentricity slows down the implementation of saliency- and relevance-driven control in time, the relative contributions of both types of control to the overall selection outcome remain unaltered, due to a concurrent overall slowing in responding.

One important question that was not addressed by van Heusden et al. (2021) is how selection control is affected when two competing stimuli are each presented at a *different* eccentricity. If eccentricity delays the effectuation of saliency- and relevance-driven control, the presentation of two competing stimuli at different eccentricities could potentially give the closer stimulus a head start relative to the one presented further away. This in turn may well create prioritized selection of the closer item, and thus change the relative impact of saliency and relevance to the ultimate selection outcome.

Indeed, there are several studies suggesting that observers prioritize the selection of less eccentric stimuli over those presented further away (Gajewski et al., 2005; Tatler et al., 2006; Van Heusden et al., 2023; Wolfe et al., 1998). In a direct test of such a central selection bias, Van Heusden et al. (2023) presented observers with displays containing two identical and equally salient target singletons, each presented at one of three possible eccentricities. Observers were asked to make an eye movement to either one of the two singletons. Their findings revealed that observers exhibited a strong bias towards selecting the target nearest to fixation. Moreover, this bias was demonstrated to be much stronger than expected on the basis of the saccade latency distributions obtained in conditions in which both targets were presented at the same eccentricity, suggesting that in competition, the presence of an eccentricity difference changes selection control. However, given that both objects were targets, and both were equally salient, Van Heusden et al. (2023) could not examine how the contributions of saliency and relevance were separately modulated by eccentricity. Moreover, none of the mentioned studies have looked at the time course of the central selection bias, and thus we know nothing about *when* during the visual selection process eccentricity exerts an influence.

The present study aims to investigate how a difference in eccentricity between competing stimuli dynamically impacts saliency- and relevance-driven selection. To do so, we used a paradigm similar to van Heusden et al. (2021; see also: Dombrowe et al., 2012; Huang et al., 2021; Siebold & Donk, 2014; van Heusden et al., 2022; Van Zoest & Donk, 2006). In this paradigm, participants are asked to make a single eye movement to an orientation singleton target that is shown simultaneously with an orientation singleton distractor embedded in a background of homogenously oriented other items. By varying the orientation of the background items across trials, the target is either more or less salient than the distractor. This paradigm enables the investigation of how the influences of saliency-driven and relevance-driven control vary across saccade latency. To examine how an eccentricity difference between singletons affects selection control, each of the singletons was presented at one of three possible eccentricities (near, middle, far), such that the eccentricity difference between them was 0 (i.e., when the singletons were presented at the same eccentricity), 1 (i.e., when the singletons were presented at different eccentricities that were one eccentricity value apart), or 2 (i.e., when the singletons were presented at different eccentricities that were two eccentricity values apart). This set-up enables us to not only investigate how the influences of saliency and relevance vary in time across eccentricity difference, but also allows us to examine the time course of a possible central selection bias.

In accordance with previous findings (e.g., Van Heusden et al., 2023; Wolfe et al., 1998), we expected to find a larger central selection bias with increasing eccentricity difference. Dependent on the time course of this central selection bias, we expected to find differential modulations of the effects of saliency and relevance. That is, if on the one hand, eccentricity biases selection primarily early on, then it should foremost affect saliency-driven selection, for saliency effects typically occur in short-latency eye movements only. If, on the other hand, the central selection bias is mainly expressed in long-latency eye movements, it should mostly affect relevance-driven selection.

## Methods

### Participants

A planned number of 33 participants participated in the experiment (age range: 18–28 years; 23 females and 10 males). All participants reported normal or corrected-to-normal vision and gave informed consent prior to participation. Participants received either course credit or a monetary reward for their participation. The protocol was approved by the ethics review board of the Faculty of Behavioral and Movement Sciences and conducted according to the tenets of the Declaration of Helsinki.

### Apparatus and stimuli

**Fig. 1:**
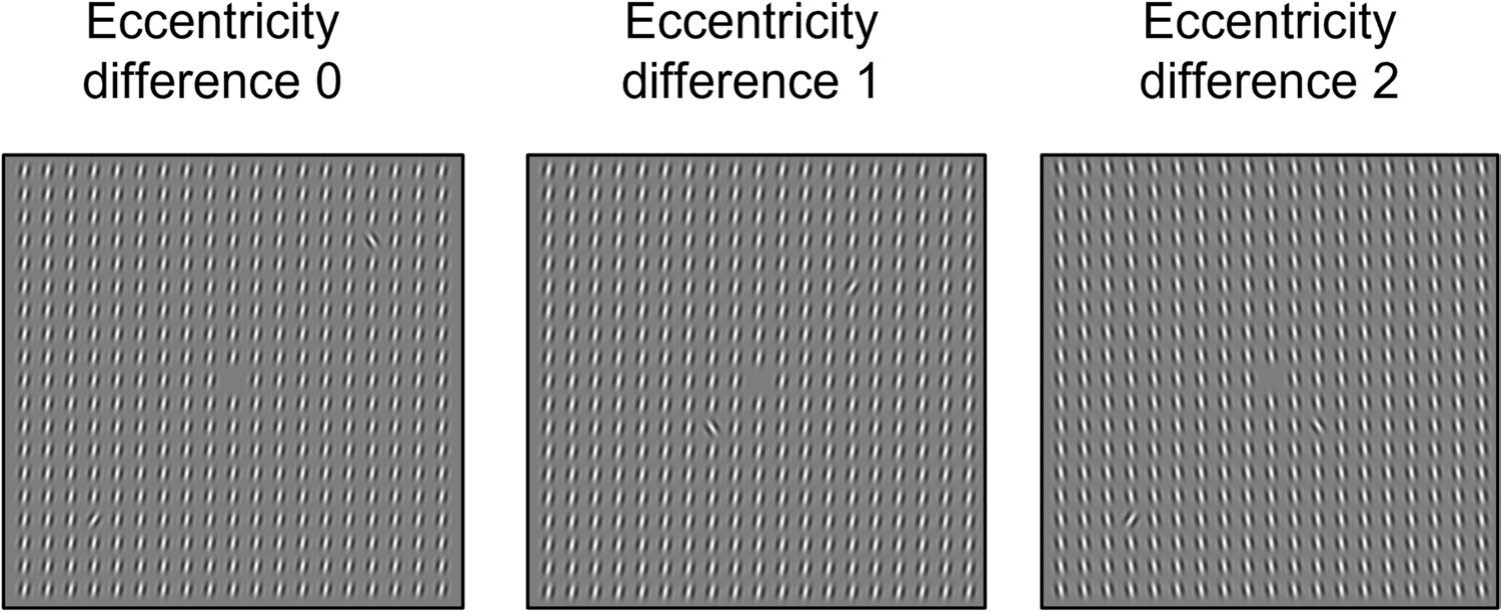
Three examples of the search display. In these examples the right-tilted singleton was the target (this was counterbalanced between participants). In the first two example displays, the background elements are tilted 10° to the right, making the 30° right-tilted singleton (the target) less salient than the 30° left-tilted singleton (the distractor). In the example display on the right, this is the other way around. In the left panel, both the target and the distractor are presented at the far eccentricity, resulting in an eccentricity difference of 0. In the middle panel, the target is presented at the middle eccentricity, while the distractor is presented at the near eccentricity. Hence, there is an eccentricity difference of 1. Lastly, in the right panel the target is presented at the far eccentricity while the distractor is presented at the near eccentricity. Thus, here the eccentricity difference is 2

Stimuli were presented on a monitor with a resolution of 1,920 × 1,080 pixels and a refresh rate of 240 Hz. Eye movements were recorded using a tower-mounted EyeLink 1000 Plus eyetracker (SR Research, Ontario, Canada). The screen was located 70 cm away from the participant with the use of a chin rest. Whenever subjects were required to fixate, a fixation cross consisting of two lines (with a stroke width of 0.07 degree of visual angle (dva), extending 0.24 × 0.24 dva) was presented. Each search display consisted of multiple homogeneously oriented background Gabors, tilted either 10° to the left or 10° to the right, and two singleton Gabors, one of which was oriented 30° to the left and the other 30° to the right. These Gabors were 1.5 dva in diameter, with a spatial frequency of 2 cycles per degree of visual angle presented at 100% contrast and were presented in a 19 × 19-element square grid (22.6 × 22.6 dva), with a center-to-center distance of 1.2 dva in both the vertical and the horizontal direction. Simultaneously presented singleton Gabors were presented on the array diagonals, each at one of three possible eccentricities, 3.4 dva (near), 6.7 dva (middle), and 10.1 dva (far) from the center of the display, such that the eccentricity difference between both singletons was 0 (i.e., when the singletons were presented at the same eccentricity), 1 (i.e., when the singletons were presented at different eccentricities that were one eccentricity value apart), or 2 (i.e., when the singletons were presented at different eccentricities that were two eccentricities values apart). On a given trial, the singletons were never presented in the same quadrant. Participants were instructed to make a speeded eye movement to a predefined target. For half of the participants the target was the left-tilted singleton, and for the other half of the participants the target was the right-tilted singleton. Depending on the orientation of the background Gabors on a given trial, the target could either be more salient than the distractor (target salient distractor non-salient trials, TSDN trials) or less salient than the distractor (target non-salient distractor salient trials, TNDS trials). Examples of the search display are presented in Fig. 1.

### Design

We used a within-subject design with Eccentricity difference (0, 1, 2) and Trial type (TSDN trials versus TNDS trials) as factors. To mitigate the potential impact of any display regularities, we presented each of the singletons equally often at each eccentricity (near, middle, far). We intended to manipulate the eccentricity of each singleton independently, such that all nine eccentricity combinations were shown an equal number of times, but due to a programming error, both singletons were presented at the same eccentricity (either near, middle, or far) on 50% rather than 33% of the trials for the first 15 participants. This was corrected for the remaining 18 participants. The first 15 participants completed a total of 1,116 experimental trials with 558 trials in which the eccentricity difference was 0, 372 trials in which the eccentricity difference was 1, and 186 trials in which the eccentricity difference was 2. The others completed a total of 1,008 experimental trials with 336 trials in which the eccentricity difference was 0; 448 trials in which the eccentricity difference was 1; and 224 trials in which the eccentricity difference was 2. Please note that the individual singletons were equally often presented at each of the possible singleton locations for all participants. As the analyses revealed no difference in the pattern of results between the two groups, we concluded that the error was not critical. The numbers of TSDN trials and TNDS trials were equal for each level of eccentricity difference. The different combinations of conditions were randomly mixed, while target identity (left-tilted or right-tilted) was counterbalanced across participants. Please note that given the presence of an eccentricity difference (i.e., in the eccentricity difference 1 and 2 conditions), the target was equally often presented at a less eccentric as at a more eccentric location. This was also true for the distractor, the most salient singleton, and the least salient singleton. As a result, the present design allows us to separate the effects of saliency, relevance, and the central selection bias for each level of eccentricity difference (e.g., eccentricity level 1), but does not allow us to examine these effects separately per unique combination of target and distractor eccentricity (e.g., target at the near eccentricity and distractor at the middle eccentricity), for in the latter case eccentricity covaries with relevance.

Prior to the experimental trials, participants first completed a practice block of 36 trials. There was a break after every 48 trials in which feedback regarding saccade latency was provided. A session took approximately 1.5 h.

### Procedure

Before the start of the experiment, a nine-point calibration was performed. Each trial started with the presentation of a central dot, required for a drift correction. After a space bar press, a central fixation cross was presented for 500 ms, followed by the search display. Subjects were instructed to fixate centrally while the fixation cross was presented and then to move their eyes toward the target singleton as soon as the search display appeared. The search display was presented without the fixation cross to encourage subjects to make a fast eye movement. The search display remained on screen until 150 ms after the eye reached an area within 1 dva from one of the two singletons. If participants failed to do so within 2,000 ms, the search display disappeared from screen.

### Data analysis

Eye-movement data were analyzed offline. Saccade start and end points were defined using the velocity-based algorithm described in Nyström and Holmqvist (2010). We calculated the saccade latency and landing position of the first saccade for each trial, where saccade latency was defined as the time between search display onset and the start of the first eye movement. Trials in which the first saccade was initiated earlier than 80 ms were discarded from further analysis. The first saccade was assigned to be directed to either one of the singletons if its landing position was located in the corresponding quadrant and was less than half of this singleton’s eccentricity away from it. Trials in which the first saccade were directed to neither the target nor the distractor were also discarded from further analyses. Saccade latency distributions were then calculated based on the remaining trials. Trials were further discarded if the saccade latency fell within the lowest 2.5% of the overall latency distribution or was greater than 500 ms.

To investigate how saccade latency was influenced by the saliency, the relevance, and the eccentricity of the selected item, we ran a repeated-measures analysis of variance (ANOVA) with Saliency of the selected item (salient, non-salient), Relevance of the selected item (target, distractor), and Eccentricity of the selected item (near, middle, or far), as factors, with α = 0.05.

To investigate how overall saliency-driven selection performance was affected by Eccentricity difference (0, 1, 2), we computed the individual averaged proportions of trials in which the eyes went to the most salient singleton (p(salient)) separately per level of Eccentricity difference (0, 1, 2). Because within each level of Eccentricity difference, the salient target, the non-salient target, the salient distractor, and the non-salient distractor were equally often presented at the least and most eccentric singleton locations, the difference between p(salient) and chance performance (.5) reflects the saliency effect. To investigate how overall relevance-driven selection performance was affected by Eccentricity difference (0, 1, 2), we computed the individual averaged proportions of trials in which the eyes went to the target (p(target)), separately for each level of Eccentricity difference (0, 1, 2). The difference between p(target) and .5 reflects the relevance effect. Similarly, to investigate whether and how selection performance was subject to a central selection bias, we computed the individual averaged proportions of trials in which the eyes went to the least eccentric singleton (p(closest)), separately for each level of Eccentricity difference.^1^ The difference between p(closest) and .5 reflects the central selection bias.

To test for the presence of a saliency effect, a relevance effect, and a central selection bias, t-tests were performed to compare p(salient), p(target), and p(closest) to chance performance (.5) separately per level of Eccentricity difference. P(salient), p(target), and p(closest) were then separately entered in a repeated-measures ANOVA with Eccentricity difference (0, 1, 2) as a factor with α = 0.05. A Greenhouse–Geisser correction was applied if the assumption of sphericity was violated (Greenhouse & Geisser, 1959).

Next, we focused on the saliency effect, the relevance effect, and the central selection bias across time. For this, we looked at changes in selection performance as a function of saccade latency, using a weighted averaging procedure described in van Leeuwen et al., (2019). First, the single-subject data were smoothed using a moving Gaussian kernel with a width of 10 ms. Then, each point in the time course (in steps of 1 ms) was assigned a weight based on the number of data points contributing to that subject's latency distribution. These weights were used to calculate weighted average performance. In doing so, this method compensates for the possibility that some subjects might have very few datapoints contributing to a certain time point. This would lead to an unreliable estimate of performance, which could distort the overall data pattern when simply averaging over participants. In order to examine the saliency effect, the relevance effect, and the central selection bias across saccade latency, we calculated the corresponding time courses of p(salient), p(target), and p(closest) in this way, separately for each level of Eccentricity difference, and tested for deviations from chance performance (.5) across the full range of saccade latency. To do so, we performed paired t-tests corrected for multiple comparisons using cluster-based permutation testing (Maris & Oostenveld, 2007) with 1,000 permutations, separately per level of Eccentricity difference. We used the same procedure to test for variations in the effects of saliency, relevance, and the central selection bias across Eccentricity difference. For a more detailed description of the procedure, see van Leeuwen et al., (2019).

## Results

Trials in which the first saccade was directed to neither the target nor the distractor (11.0 %) and those in which the saccade latency fell outside our latency criteria (5.9%; see *Methods*) were discarded from further analyses.

### Saccade latency

**Fig. 2:**
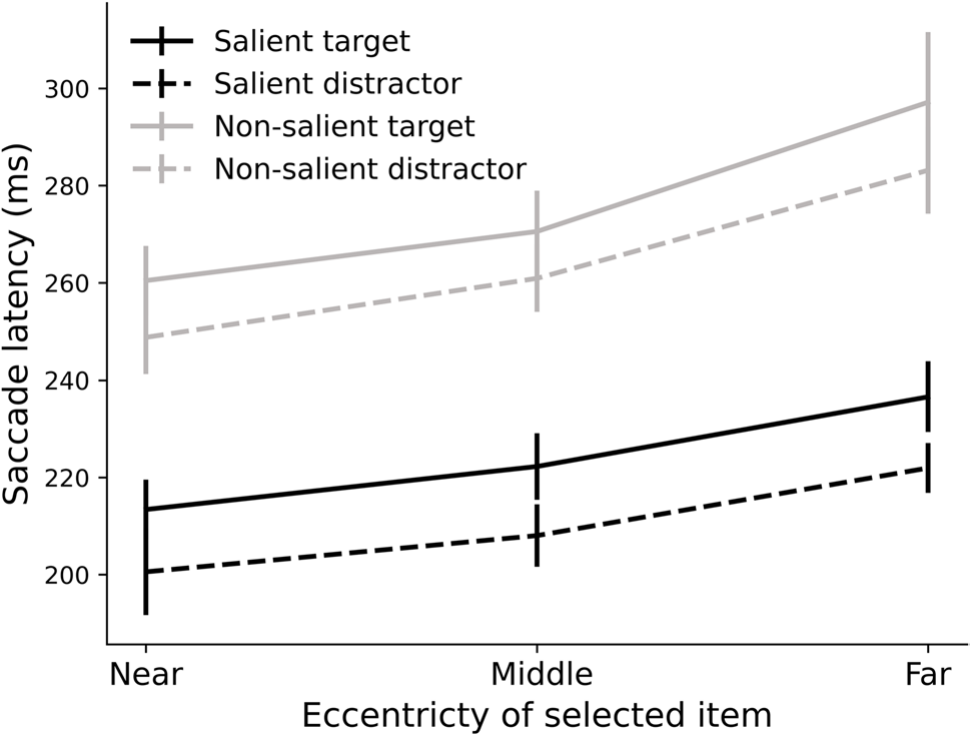
Average saccade latency in milliseconds as a function of eccentricity of selected item (near, middle, and far) plotted separately for the four possible items to be selected: salient target, salient distractor, non-salient target, and non-salient distractor. All error bars reflect 95% within-subject confidence intervals (cf. Cousineau, 2005)

Figure 2 depicts the averaged saccade latency as a function of the eccentricity of the selected item, separately for trials in which the selected item was a salient target, a salient distractor, a non-salient target, or a non-salient distractor. We conducted a repeated-measures ANOVA with the factors Saliency of the selected item (salient, non-salient), Relevance of the selected item (target, distractor), and Eccentricity of the selected item (near, middle, far). Four participants had no observations in at least one of the cells and were therefore not included in the ANOVA and in the data of Fig. 2. This exclusion did not visually change the pattern of the data. This analysis revealed a main effect of Saliency of the selected item (F(1,28) = 147.19, p < 0.001, η_p_^2^ = 0.84), as saccades to salient elements were initiated quicker than saccades to non-salient elements. The main effect of Relevance of the selected item was significant too (F(1,28) = 11.21, p < 0.01, η_p_^2^ = 0.29) as participants were faster to make a saccade to a distractor than to a target. This may seem counterintuitive, but this is in line with previous findings showing that the rapid saccades tend to be the more erroneous ones (i.e., to a distractor; we will observe this later as well). We also observed a main effect of Eccentricity of the selected item (F(1.39,38.97) = 30.11, p < 0.001, η_p_^2^ = 0.52). Pairwise comparisons revealed that all contrasts were significant (all F-values > 8.46, all p-values < 0.01, all η_p_^2^ > 0.23), confirming that saccade latency increased with each level of Eccentricity of the selected item. Lastly, there was a significant interaction between Saliency and Eccentricity of the selected item (F(1.58,44.26) = 3.70, p < 0.05, η_p_^2^ = 0.12), reflecting that the eccentricity-dependent increase in saccade latency was larger for non-salient than for salient items. The other interaction effects did not reach significance (all F-values < 0.28, all p-values > 0.60). Together, these findings show that eccentricity modulated the effect of saliency on saccade latency, but not that of relevance.

### Selection behavior

**Fig. 3:**
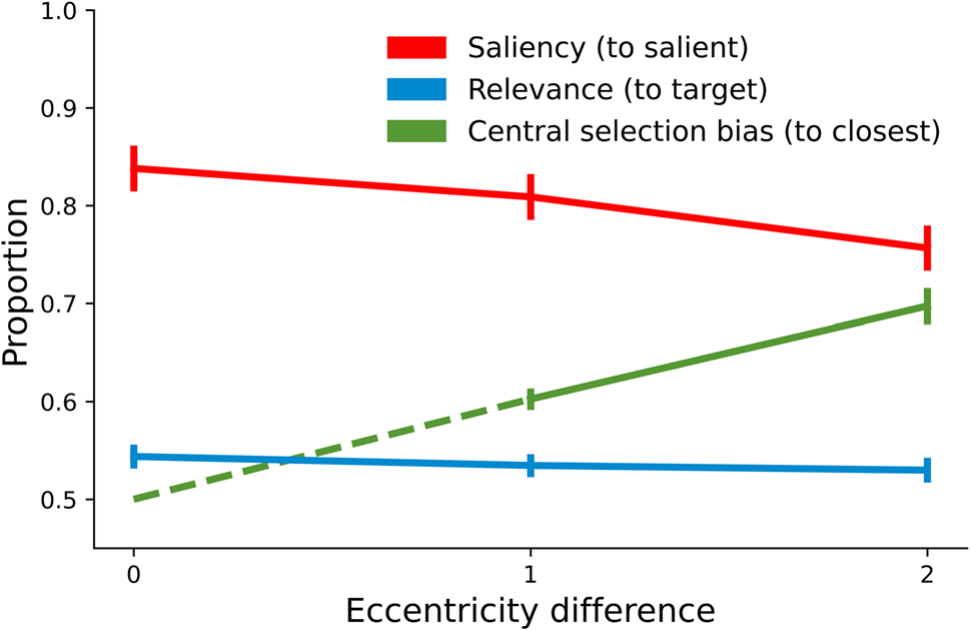
Proportion of trials in which the most salient item was selected (red), the most relevant item was selected (blue), and the least eccentric item was selected (green) as a function of Eccentricity difference. Error bars reflect standard error. Please note that the dashed green line between Eccentricity differences 0 and 1 serves to signify that the data point at Eccentricity difference 0 is theoretically specified (see also Footnote 1)

Figure 3 depicts the proportion of trials in which the most salient singleton was selected (p(salient), thus reflecting the *saliency effect*), the most relevant singleton was selected (p(target), thus reflecting the *relevance effect*), and the least eccentric singleton was selected (p(closest), thus reflecting the *central selection bias*), as a function of Eccentricity difference. All proportions (apart from p(closest) in the Eccentricity difference 0 condition) are significantly larger than .5 (all ps < 0.03). To investigate the effect of eccentricity difference on the saliency effect we performed a repeated-measures ANOVA on p(salient) with Eccentricity difference (0,1,2) as its only factor. This revealed a main effect of eccentricity difference (F(1.26, 40.43) = 37.05, p < 0.001, η_p_^2^ = 0.54), reflecting the fact that the effect of saliency decreased with increasing eccentricity difference between both singletons. A similar ANOVA on p(target) revealed no such effect (F(2,64) = 2.36, p = 0.1). In other words, the effect of relevance was not reliably modulated by Eccentricity difference. Finally, a repeated-measures ANOVA on p(closest) with Eccentricity difference (1,2) as its only factor revealed a main effect of eccentricity difference (F(1, 32) = 98.48 p < 0.001, η_p_^2^ = 0.76), reflecting the fact that observers were more likely to select a close over a far item when Eccentricity difference increased.^2^ Overall, these results show a larger central selection bias with increasing eccentricity difference. Importantly, this goes along with a simultaneous reduction of the saliency effect, while the relevance effect remains intact.

### The saliency effect, relevance effect, and central selection bias as a function of time

**Fig. 4:**
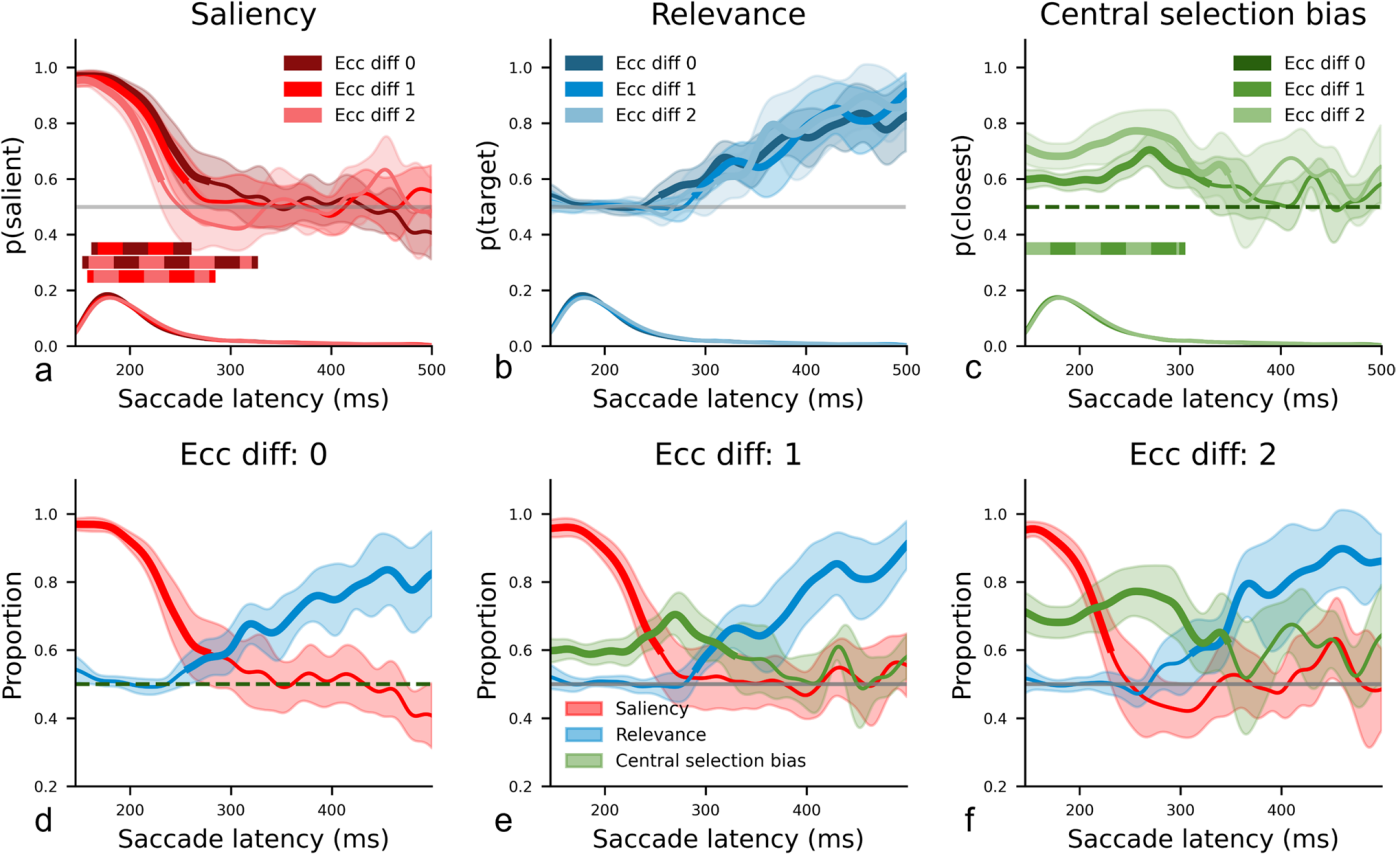
**a*–*c**: Time courses of the proportion of trials in which the most salient (**a**), the most relevant (**b**), and the least eccentric (**c**) singleton was selected as a function of eccentricity difference. Bold lines indicate where performance differed significantly from chance performance. Shaded areas correspond to 95% confidence intervals. The horizontal bars denote all time points where there is a significant difference between conditions, with the alternating colors specifying the conditions being compared. The corresponding probability density estimations of the underlying saccadic latency distributions are depicted at the bottom of each plot. **d*–*f**: The time courses from a*–*c combined in separate panels for each level of Eccentricity difference

Figure 4a shows the time courses of the proportion of trials in which the most salient item was selected, plotted separately for each level of Eccentricity difference. Replicating previous findings, we show that when both singletons were presented at the same eccentricity (Eccentricity difference 0), early eye movements were mainly saliency driven, as p(salient) was initially significantly larger than chance. Similar to overall performance, this saliency effect was strongest when the two singletons were presented at the same eccentricity (Eccentricity difference 0) and declined with increasing eccentricity difference between both singletons.

Figure 4b shows the time courses of the proportion of trials in which the most relevant item was selected, plotted separately for each level of Eccentricity difference. The results show that p(target) started to significantly deviate from chance performance at about 250–300 ms, and rose from thereon. Overall, Eccentricity difference did not modulate the effect of relevance.

Figure 4c shows the time courses of the proportion of trials in which the least eccentric singleton was selected, plotted separately for each level of Eccentricity difference. As is evident from these time courses, there was a strong central selection bias, as p(closest) deviated significantly from chance performance. Importantly, this bias was limited to early eye movements only and disappeared with increasing saccade latency. Overall, this central selection bias increased with increasing eccentricity difference.

Figures 4d–f depict the saliency effect, the relevance effect, and the central selection bias arranged separately per level of eccentricity difference. While the central selection bias (bold green lines) increased with increasing eccentricity difference, the effect of saliency (bold red lines) decreased. That is, the effect of saliency became increasingly overruled by the central selection bias as the eccentricity difference increased. Both effects were particularly pronounced in the early time window, although the time course of the central selection bias extended beyond the one of the saliency effect. The effect of relevance (bold blue lines) occurs later and was little affected by eccentricity difference. Together, these results show that the presence of an eccentricity difference between two competing items substantially affected selection, such that the eyes were less affected by saliency and more by the tendency to select the most central item.

## Discussion

Research on the control of visual selection (Folk et al., 1992; Itti & Koch, 2000; Theeuwes, 1992, 1994) has traditionally focused on the question of whether selection behavior is driven by saliency or relevance. The debate has largely ignored the possibility that these influences might well be modulated, or even overruled by eccentricity-based biases. Including eccentricity in models of selection is important, as several studies have demonstrated that in competition, less eccentric items are prioritized for selection over more eccentric ones, suggesting the existence of a central selection bias (Van Heusden et al., 2023; Wolfe et al., 1998). Yet, such a bias has never been considered to play any role of significance in how saliency and relevance affect the selection outcome. Here we investigated whether observers exhibit a central selection bias under conditions in which competing items differ in saliency and relevance, and how this affects selection. We demonstrated that observers prioritize the selection of less eccentric over more eccentric items. This central selection bias increases with increasing eccentricity difference between competing items, to the extent that observers select the less eccentric singleton more than twice as often as the more eccentric one. Overall, the effect of saliency is reduced when the eccentricity difference between competing singletons increases, whereas the effect of relevance remains unaltered. In other words, the presence of a central selection bias attenuates the effect of saliency but not of that of relevance. Indeed, our time-course analyses show that the central selection bias and saliency effects occur within the same time window, in that both effects happen relatively early and only transiently after the presentation of the search display, and a stronger central selection bias with increasing eccentricity difference goes together with reduced effects of saliency. This suggests that the central selection bias is not an isolated phenomenon, but fundamentally reduces the relative contribution of saliency to selection control. In contrast, the effects of relevance are not affected by eccentricity difference. The time course of the relevance effect proved to be rather different not only from the saliency effect, but also from that of the central selection bias. Relevance affects selection relatively late, well beyond the time window in which the central selection bias occurs, and consequently neither its onset in time nor its size is modulated by eccentricity difference.

The finding that the central selection bias is only operational in a finite time window (i.e., until approximately 350 ms) is inconsistent with the idea that it represents a general motor bias in the oculomotor system that promotes small over large eye movements (Bahill et al., 1975; Collins et al., 1975; Gajewski et al., 2005; Tatler, 2007; Wang & Hsiang, 2011). Such a motor bias would have been expected to occur across the full time range of saccade latencies. Our results show that this was not the case. Neither does the central selection bias seem to be an expression of a top-down strategic preference derived from expectations regarding the probable target location (Feng & Spence, 2013; Laberge & Brown, 1989). In the present study, the target was presented with equal probability at each eccentricity, and thus any strategic choice here would have been pointless. More importantly, previous studies have demonstrated that strategic, endogenous allocation of spatial attention takes about 300 ms or more, and is typically sustained for even longer (Cheal & Lyon, 1991; Muller & Rabbitt, 1989; Posner & Cohen, 1984). The present results show that the central selection bias occurs rapidly and its time course is transient, suggesting that it is not endogenously driven. In this context it is important to mention that there are in fact studies that suggest that observers show a strategic preference to fixate locations close to the center of an image (Clarke & Tatler, 2014; Peacock et al., 2020; Rothkegel et al., 2017; Schutt et al., 2019; Tatler, 2007, 2009; Tatler & Vincent, 2009; Tseng et al., 2009). Various studies on natural image viewing have demonstrated that the eyes show a strong preference to (initially) move to the center of an image rather than to its peripheral parts. This central fixation bias, which needs to be distinguished from the central selection bias here, refers to a bias relative to the center of a (natural) image and not to a bias relative to fixation, and is often considered to be, at least to a certain extent, strategic in nature. Indeed, in pictures of natural images, the most interesting objects are often located in the center of the picture, and the tendency to preferably fixate those parts may well be driven by this knowledge (Parkhurst et al., 2002; Tatler, 2007, 2009; Tseng et al., 2009). Moreover, the center of a natural image is also regarded as the most optimal location for gist extraction (Tatler, 2007; Torralba et al., 2006), which in turn may further contribute to a strategic preference to fixate central rather than more peripheral image locations. However, even though the initial fixation point was positioned in the center of the screen in the present study, it is unlikely that our results were shaped by a strategic preference towards the center of the image because there was no need to extract scene information but rather to make a single goal-directed eye movement to a prespecified target. More importantly, the present results basically show that the central selection bias already occurs for the earliest eye movements, well before the typical period in which possible strategic control can be expressed (see also Wolf & Lappe, 2021).

One way to account for the central selection bias in the present study is by postulating that eccentricity modulates the speed at which information becomes available for visual selection. It is well documented that increasing the eccentricity of a visual target increases reaction time and decreases accuracy (Carrasco et al., 1995; Carrasco et al., 1998; Staugaard et al., 2016), suggesting that the speed of visual processing decreases with increasing eccentricity. If less eccentric items are processed faster than more eccentric ones, then the former items are available for selection at an earlier point in time than the latter ones. Accordingly, during a limited period of time after the presentation of the search display, selection priority will be biased towards less eccentric items solely because the more distant items have not yet been (fully) processed. This notion of the central selection bias shows much similarity to our dynamic notion of saliency effects (Donk & van Zoest, 2008, 2011; Siebold et al., 2011; van Heusden et al., 2021; Van Heusden et al., 2023; van Heusden et al., 2022; van Zoest & Donk, 2005, 2006, van Zoest & Donk, 2008). According to this view, salient items are not prioritized over less salient ones because they generate more activity in the priority map of selection (Fecteau & Munoz, 2006; Itti & Koch, 2001; Luck et al., 2021) but merely because they generate activity at an earlier point in time. Thus, rather than taking saliency as a factor that continuously affects visual selection, its effects are merely perceived as a by-product of the differential speeds with which individual items are made available in the priority map (Donk & Soesman, 2010, 2011). Here we propose that eccentricity affects selection control in a similar way by suggesting that less eccentric items shape the priority map at an earlier point in time than more eccentric ones. Such an account would not only explain the short-lived nature of the central selection bias but also its co-occurrence in time with the effects of saliency. If both a difference in eccentricity and a difference in saliency between two items modulate the time at which these items become available for selection, then both should affect selection early on and both effects should be transient, which was indeed the case. More importantly, such an account also explains the interplay between eccentricity and saliency as evident from the interaction between eccentricity and saliency in the saccade latency results (Fig. 2), overall selection behavior (Fig. 3), and the temporal modulations in the saliency effects across eccentricity difference (Fig. 4). If eccentricity and saliency both affect when items are available in the priority map, changing one should also modulate the effect of the other.

It is important to note that a number of studies suggest that the speed of processing increases rather than decreases from the center of vision to the periphery (Carrasco et al., 2003; Carrasco et al., 2006; Upadhyayula et al., 2023). However, in these studies stimuli were typically presented only very briefly. For instance, in a study conducted by Carrasco et al. (2003), stimulus displays were presented for 40 ms, while our stimuli remained visible until one of the singletons was selected. It might well be that briefly presented, transient stimuli undergo faster processing at greater eccentricities while the processing of more static stimuli, as used in the present study, is compromised. Indeed, the processing of dynamic stimuli has been linked to a greater engagement of the magnocellular system whereas the processing of static stimuli more strongly relies on the parvocellular system (Carrasco et al., 2003; Carrasco et al., 2006). Magnocellular cells exhibit larger speeds of conduction than parvocellular cells (Lamme & Roelfsema, 2000; Schmolesky et al., 1998), and the number of magnocellular cells increases with eccentricity while the number of parvocellular cells decreases (Azzopardi et al., 1999). Hence, the results from these earlier studies involving dynamic stimuli do not necessarily contradict the results observed here but rather emphasize, as we do, the significance of eccentricity as a key factor in visual search.

Traditional models of selection control typically do not take into account the possibility that selection control changes across time and space (Folk et al., 1992; Gaspelin & Luck, 2018; Itti et al., 1998; Itti & Koch, 2000; Theeuwes, 1992, 1994; Torralba et al., 2006; Wolfe, 1994; Zhang et al., 2019). However, the results of the present study show the need for incorporating both these factors in models of selection control. There are several theoretical approaches that actually do take temporal and spatial variations into account to explain visual search behavior (Buetti et al., 2016; Geisler & Chou, 1995; Hulleman & Olivers, 2017; Lleras et al., 2022; Wolfe, 2021; Zelinsky, 2008). However, these models typically focus on either saliency (Geisler & Chou, 1995) or relevance (Buetti et al., 2016; Zelinsky, 2008) without incorporating both sources of control. Moreover, the temporal dynamics that are taken into account are typically limited to changes in relation to changes in the position of the eye relative to the visual information. That is, in these models selection is assumed to depend on the eccentricity at which information is presented, and selection control might well change over time but only because the eyes typically change position during visual search, resulting in dynamic changes in visual input. Temporal changes in the underlying control processes of selection within a single fixation (i.e., across saccade latency) are usually not included.

Taken together, the present results show that the size of an eccentricity difference between competing items modifies the earliest responses of selection. This changes the relative contribution of saliency but not of that of relevance. Together, these results suggest that selection control is subject to change, not only across time but also across space.

## Footnotes

1 Please note that target and distractor were presented at the same eccentricity in the Eccentricity difference 0 condition. To allow for comparison between the central selection bias and the effects of saliency and relevance across the different levels of Eccentricity difference, we set the proportion in this condition equal to chance level (.5).

2 Note that in trials where the eccentricity difference is 2, the closest singleton consistently appears at the near eccentricity, while this occurs in only half of the trials when the eccentricity difference is 1. Possibly then, the pattern of results is driven by the absolute rather than the relative eccentricity of the nearest singleton. To investigate this, we categorized trials in the eccentricity difference 1 condition into two groups: near-middle trials, where one singleton was presented at the near eccentricity and the other at the middle eccentricity, and middle-far trials, where one singleton was presented at the middle eccentricity and the other at the far eccentricity. We found that observers are somewhat more likely to select the salient singleton (.82 vs. .80; t(32) = 3.42, p = 0.002) and less likely to select the closest singleton (.58 vs. .62; t(32) = -2.94, p = 0.006) in the near-middle as compared to the middle-far trials, while there is no effect for relevance (.53 vs. .54; t(32) = -1.14, p = 0.262). Importantly, the overall pattern of results remains unchanged regardless of whether only the near-middle or middle-far trials are included. This goes for the decline in the saliency effect with eccentricity difference (ps < 0.001), the increase in central selection bias with increasing eccentricity difference (ps < 0.001), as well as the null effect for relevance (ps > 0.091).
